# Effect of Toxicants on Fatty Acid Metabolism in HepG2 Cells

**DOI:** 10.3389/fphar.2018.00257

**Published:** 2018-04-23

**Authors:** David Grünig, Urs Duthaler, Stephan Krähenbühl

**Affiliations:** ^1^Division of Clinical Pharmacology and Toxicology, University Hospital Basel, Basel, Switzerland; ^2^Department of Biomedicine, University of Basel, Basel, Switzerland; ^3^Swiss Centre for Applied Human Toxicology, Basel, Switzerland

**Keywords:** liver steatosis, fatty acids, biomarkers, ApoB-100, acylcarnitines, dicarboxylic acids

## Abstract

Impairment of hepatic fatty acid metabolism can lead to liver steatosis and injury. Testing drugs for interference with hepatic fatty acid metabolism is therefore important. To find out whether HepG2 cells are suitable for this purpose, we investigated the effect of three established fatty acid metabolism inhibitors and of three test compounds on triglyceride accumulation, palmitate metabolism, the acylcarnitine pool and dicarboxylic acid accumulation in the cell supernatant and on ApoB-100 excretion in HepG2 cells. The three established inhibitors [etomoxir, methylenecyclopropylacetic acid (MCPA), and 4-bromocrotonic acid (4-BCA)] depleted mitochondrial ATP at lower concentrations than cytotoxicity occurred, suggesting mitochondrial toxicity. They inhibited palmitate metabolism at similar or lower concentrations than ATP depletion, and 4-BCA was associated with cellular fat accumulation. They caused specific changes in the acylcarnitine pattern and etomoxir an increase of thapsic (C18 dicarboxylic) acid in the cell supernatant, and did not interfere with ApoB-100 excretion (marker of VLDL export). The three test compounds (amiodarone, tamoxifen, and the cannabinoid WIN 55,212-2) depleted the cellular ATP content at lower concentrations than cytotoxicity occurred. They all caused cellular fat accumulation and inhibited palmitate metabolism at similar or higher concentrations than ATP depletion. They suppressed medium-chain acylcarnitines in the cell supernatant and amiodarone and tamoxifen impaired thapsic acid production. Tamoxifen and WIN 55,212-2 decreased cellular ApoB-100 excretion. In conclusion, the established inhibitors of fatty acid metabolism caused the expected effects in HepG2 cells. HepG cells proved to be useful for the detection of drug-associated toxicities on hepatocellular fatty acid metabolism.

## Introduction

Hepatocellular metabolism of fatty acids is a complex function of hepatocytes. During starvation, when fatty acids become the most important fuel, activated fatty acids are mainly transported into the mitochondrial matrix for subsequent β-oxidation (Soeters et al., 2012). After ingestion of a meal, the high insulin concentration blocks transport of fatty acids into the mitochondrial matrix by inhibiting carnitine palmitoyltransferase (CPT) 1A, thereby shifting activated fatty acids into triglyceride synthesis (Foster, 2012). Triglycerides can be stored in lipid vesicles or be excreted as very low-density lipoprotein (VLDL).

Several drugs have been shown to interfere with hepatic metabolism of fatty acids. Well-known examples are for instance valproic acid, tamoxifen, amiodarone and methotrexate (Fromenty and Pessayre, 1995; Amacher, 2014; Ress and Kaser, 2016). Since interference with hepatic fatty acid metabolism can be associated with liver injury, possible effects on fatty acid metabolism of drug candidates should be assessed in the preclinical and clinical phases of drug development. Fatty acid oxidation can be determined reliably in isolated liver mitochondria, cultured hepatocytes and in experimental animals (Krahenbuhl et al., 1994; Knapp et al., 2008; Felser et al., 2013). The methods usually used involve metabolism of radioactive substrates as well as spectrophotometric or radioenzymatic determination of enzyme activities. These methods are time-consuming and complicated and therefore difficult to use as biomarkers in cell cultures, experimental animals or humans. In order to be able to detect the potential for interference with hepatic lipid metabolism early, suitable cell models and, importantly, biomarkers that can also be used *in vivo*, are necessary.

Hepatic mitochondrial fatty acid breakdown starts with the activation of a given fatty acid at the outer mitochondrial membrane and ends with the formation of acetyl-CoA (and propionyl-CoA from odd-chain fatty acids) in the mitochondrial matrix. When this process is impaired, substrates proximal to the affected enzyme or transporter accumulate within the hepatocyte. After the initial activation step, accumulating substrates are acyl-CoAs of different chain lengths of the acyl group, depending on the location of the block. Since acyl-CoAs are too polar for crossing biological membranes by diffusion and efficient transport systems do not exist, they cannot leave hepatocytes and do not reach the blood. Acyl-CoAs can be converted to the corresponding acylcarnitines, however, which can be transported out of mitochondria and hepatocytes and reach the blood. This is well-established in patients with defects in hepatic fatty acid oxidation such as medium-chain acyl-CoA dehydrogenase deficiency, who typically have elevated medium-chain acylcarnitine plasma and urine concentrations (Minkler and Hoppel, 1993; Vernez et al., 2003). Acylcarnitines in plasma or urine of patients or in the supernatant of cell cultures could therefore be used as biomarkers for impaired hepatic β-oxidation. In addition, when mitochondrial β-oxidation of fatty acids is impaired, cytochrome P450 (CYP) 4A1 (CYP4A11 in humans) is induced, which catalyzes the formation of dicarboxylic acids (Kaikaus et al., 1993; Wanders et al., 2011). Dicarboxylic acids represent therefore also a potential biomarker for detecting inhibition of hepatic fatty acid metabolism in cell cultures and *in vivo*.

The principle aims of the current study were to investigate the effect of different compounds known to be associated with liver steatosis on a human hepatocyte cell line, to find out underlying mechanisms and to assess the suitability of the different assays used to determine fatty acid metabolism. Based on our own experience (Felser et al., 2013) and on reports from others (Guo et al., 2006; Donato et al., 2009), we decided to use HepG2 cells. HepG2 cells are a hepatoma cell line used widely for toxicological studies. They are easy to culture and are well-suited for studying mitochondrial functions (Felser et al., 2014a; Kamalian et al., 2015), but have only a minor expression of hepatocyte specific functions such as for instance expression of CYP enzymes (Berger et al., 2016). In the current study, we first investigated the effect of three established inhibitors of hepatic fatty acid metabolism on different aspects of fatty acid metabolism in HepG2 cells (see Supplementary Figure 1 for chemical structures). In a second step, we used the findings obtained in the first part of the study to investigate the effect of three compounds (amiodarone, tamoxifen, and WIN 55,212-2; all of them known to cause hepatocellular triglyceride accumulation) on fatty acid metabolism in HepG2 cells.

## Materials and Methods

### Chemicals

4-bromocrotonic acid (4-BCA) was purchased from TCI Chemicals (Eschborn, Germany). WIN 55, 212-2 was purchased from Cayman Chemical (Ann Arbor, MI, United States). Etomoxir, methylenecyclopropylacetic acid (MCPA), amiodarone, and tamoxifen were obtained from Sigma-Aldrich (Buchs, Switzerland). If not stated otherwise, all other chemicals were also purchased from Sigma-Aldrich (Buchs, Switzerland). Stock solutions were prepared in DMSO and kept at -20°C for cell treatment (in general at 1:1000 dilution).

### Cell Culture

The human hepatocellular carcinoma cell line HepG2 was purchased from ATCC (Molsheim Cedex, France). Cells were cultured in Dulbecco’s modified Eagle low glucose medium (1 g/L) (DMEM) supplemented with 10% (v/v) heat inactivated fetal bovine serum (FBS), 1% 1 M HEPES buffer, 1% 200 mM GlutaMAX^TM^-I supplement and 1% MEM non-essential amino acids (100×). Cells were kept in a humidified incubator at 37°C and 5% CO_2_. TrypLE express trypsin was used for passaging cells and a Neubauer hemacytometer for cell counting.

### Plasma Membrane Integrity

Plasma membrane integrity was assessed by the detection of adenylate kinase (AK) release into supernatant of the incubations. AK was determined with the ToxiLight assay kit (Lonza, Basel, Switzerland) and as described previously (Felser et al., 2013).

### Intracellular ATP Content

The intracellular ATP content was measured using the CellTiter-Glo Luminescent cell viability assay (Lonza, Basel, Switzerland) according to the manufacturer’s protocol as described previously (Felser et al., 2013).

### Protein Content

We assessed the protein content of the samples after dissolving the cells in RIPA-buffer (50 mM Tris–HCl, 150 mM NaCl, 1% Triton X-100, 0.5% sodium deoxycholate, 0.1% sodium dodecyl sulfate and 1 mM EDTA in MilliQ water, pH 7.4) using the Pierce BCA Protein Assay Kit (Thermo Scientific, Wohlen, Switzerland) according to the manufacturer’s instructions. Samples were measured at 562 nm using the Tecan plate reader.

### Intracellular Lipid Accumulation

We used the method described by Donato et al. (2009) with some modifications. HepG2 cells were incubated in the presence of drugs and exogenous lipids (DMEM containing 500 μM of a 2:1 mixture of oleate and palmitate). Oleate and palmitate were kept as stock solutions containing 667 μM oleate or 333 μM palmitate in DMEM containing 1% fatty acid free bovine serum albumin. After 24 h exposure, cells were detached using Trypsin-EDTA (Thermo Scientific, Wohlen, Switzerland) and washed with PBS. Afterwards, cells were stained with 10 μg/mL propidium iodide to exclude dead cells and 250 ng/mL BODIPY 493/503 (Thermo scientific, Wohlen, Switzerland) in PBS for 30 min at 37°C. Stained cells were examined by flow cytometry.

### Fluorescence Microscopy

HepG2 cells were seeded in black corning CellBIND 96 well assay plates with clear flat bottom purchased from Corning Inc. (Corning, NY, United States). After treatment with the toxicants, the cells were washed and fixed for 4 min with 4% paraformaldehyde solution. After an additional washing step, the cells were stained for 30 min with 0.5 μg/mL BODIPY 493/503 Thermo Scientific (Wohlen, Switzerland) and 1 μM of DAPI Thermo Scientific (Wohlen, Switzerland) in PBS. After washing twice with PBS and addition of antifade-mounting medium (1 mg/mL p-phenylenediamine in 90% glycerol, pH 9), cells were imaged using an Olympus IX83 microscope (Olympus, Tokyo, Japan).

### β-Oxidation of ^14^C-Palmitate

Metabolism of 1-^14^C-palmitic acid (60 mCi/mmol; PerkinElmer, Schwerzenbach, Switzerland) was determined as the formation of ^14^C-acid-soluble β-oxidation products in HepG2 cells treated for 24 h with test compounds. Measurements were performed as previously described (Kaufmann et al., 2005) with some modifications. HepG2 cells were permeabilized (10 μg digitonin/million cells), incubations contained 2 mM 1-^14^C-palmitate (10 nCi/assay) and the incubation lasted 15 min.

### Mitochondrial Isolation

Male C57BL/6 mice were kept in the animal facility of the University Hospital Basel (Basel, Switzerland) in a temperature-controlled environment with a 12-h light/dark cycle and food and water *ad libitum*. Animal procedures were conducted in accordance with the institutional guidelines for the care and use of laboratory animals. Mice were sacrificed by cervical dislocation. Before the liver was removed, it was flushed with 5 mL of ice cold isolation buffer (200 mM mannitol, 50 mM sucrose, 1 mM Na_4_EDTA, 20 mM HEPES, pH 7.4) and then immersed in ice-cold isolation buffer. Mitochondria were isolated by differential centrifugation as described by Hoppel et al. (1979). The mitochondrial protein content was determined using the Pierce BCA Protein Assay Kit (Thermo Scientific, Wohlen, Switzerland).

### Activity of Carnitine Palmitoyltransferase (CPT) 1

Carnitine palmitoyltransferase 1 activity was assessed by the formation of palmitoyl-^14^C-carnitine from palmitoyl-CoA and ^14^C-carnitine as described previously (Felser et al., 2014b). One hundred and twenty-five micrograms of previously frozen mitochondrial protein was the enzyme source. The substrate concentrations were 400 μM ^14^C-L-carnitine (25 pCi per assay) and 200 μM palmitoyl-CoA. The reaction was followed for 10 min.

### Mitochondrial Metabolism of Palmitoylcarnitine

The metabolism of 1-^14^C palmitoylcarnitine was assessed as described by Felser et al. (2014b). Freshly isolated mitochondria (125 μg mitochondrial protein) were incubated with 200 μM [1-^14^C]-palmitoylcarnitine (25 pCi per assay). The reaction was stopped after 4 min.

### Activity of Acyl-CoA Dehydrogenases

The activity of the respective acyl-CoA dehydrogenase was determined by the method of Hoppel et al. (1979). In a 24-well plate, 50 μg of previously frozen mitochondrial protein was preincubated in the presence of test compounds for 3 min in assay buffer (34 mM potassium phosphate, 1.5 mM KCN, 3.75 μM rotenone, 1.5 mM cytochrome C, 3 mM phenazine ethosulfate, pH 7.2). The reaction was started by the addition of palmitoyl-CoA or octanoyl-CoA (final concentrations were 50 and 100 μM, respectively) and monitored at 550 nm over 2 min using a Tecan plate reader.

### Acylcarnitines

Acylcarnitines were analyzed by liquid chromatography tandem mass spectrometry (LC-MS/MS) according to Morand et al. (2013) with some adaptions. In brief, the supernatants of cells were mixed with internal standard solution containing acetylcarnitine-d3 (10 μM), octanoylcarnitine-d3 (1 μM), and palmitoylcarnitine-d3 (1 μM) in methanol in a ratio of 1:3 (v/v). The mixture was centrifuged at 3220 ×*g* for 30 min (Eppendorf Centrifuge 5810R) and the supernatant transferred to an autosampler tube and diluted 1:1 with water before analysis. The LC-MS/MS system consisted of a Nexera SIL-30AC autosampler, a column-oven (CTO-20A), four HPLC pumps (2× LC-20AD and 2× LC-ADXR) and a system controller (CBM-20A), all acquired from Shimadzu (Kyoto, Japan). The HPLC system was coupled to an API 4000 triple quadrupole mass spectrometer from AB Sciex (Concord, Canada), equipped with a turbo electrospray ionization source.

Samples were separated on a Luna C8 5 μM column (150 mm × 2 mm) using a C8 (4 mm × 2.0 mm) precolumn (Phenomenex, Torrance, CA, United States) at 50°C. Mobile phase A was an aqueous solution of 5 mmol/L heptafluorobutyric acid and 6 mmol/L ammonium acetate. Mobile B was methanol with the same additives. Samples were loaded onto the analytical column using 20% mobile phase B. After 0.5 min, the gradient was linearly increased within 3 min to 95% mobile phase B. The column was washed for 2 min at 95% mobile phase B and thereafter reconditioned for another 0.5 min at 20% mobile phase B. The retention times of acetylcarnitine, C3-carnitine, C4-carnitine, C5-carnitine, C6-carnitine, C8-carnitine, C10-carnitine, C12-carnitine, C14-carnitine, palmitoylcarnitine, and 3-oxo-palmitoylcarnitine were 1.24, 1.44, 2.09, 2.48, 2.82, 3.18, 3.45, 3.64, 3.82, 3.98, and 3.78 min, respectively.

Acylcarnitines of interest were analyzed in the positive mode by multiple reaction monitoring (MRM). Acylcarnitines build a characteristic fragment of 85 m/z, which corresponds to a McLafferty rearrangement of the butyric acid side chain with loss of the trimethylamine moiety (Zuniga and Li, 2011). The following mass transitions (m/z) were used: acetylcarnitine, 204→85; acetylcarnitine-d3, 207→85; C3-carnitine, 218→85; C4-carnitine, 232→85; C5-carnitine, 246→85; C6-carnitine, 260→85; C8-carnitine, 288→85; C8-carnitine-d3, 291→85; C10-carnitine, 316→85; C12-carnitine, 344→85; C14-carnitine, 372→85; palmitoylcarnitine, 400→85; palmitoylcarnitine-d3, 403→85; 3-oxo-palmitoylcarnitine, 414→85. Reference substances were obtained for acetylcarnitine, octanoylcarnitine, and palmitoylcarnitine and used as standards and for quality control. The ion spray voltage was 5,500 eV, the probe temperature was 450°C, and the dwell time was 20 ms for each analyte.

### Dicarboxylic Acids

After treatment of the cells with the toxicants in 12-well plates, the cell suspensions (500 μL) were frozen and thawed before the addition of 500 μL of internal standard solution (methanol containing 1 μM sebacic acid-d16). Samples were diluted further with 1000 μL internal standard solution for protein precipitation and then centrifuged at 15,500 ×*g* for 10 min at 15°C. The supernatant was transferred to an autosampler tube and analyzed using the LC-MS/MS system described for the analysis of acylcarnitines. Samples were separated on a Symmetry C18 3.5 μM (4.6 mm × 75 mm) column (Waters Corporation, Milford, MA, United States). Mobile phase A was water containing 0.1% formic acid and mobile B was methanol containing 0.1% formic acid. Samples were loaded onto the analytical column using 50% mobile phase B. After 0.25 min, the gradient was linearly increased within 1.25 min to 95% mobile phase B. The column was washed for 1.5 min at 95% mobile phase B and thereafter reconditioned for another 0.5 min at 50% mobile phase B. The retention times of suberic acid, sebacic acid, and thapsic acid were 1.35, 1.78, and 2.45 min, respectively. Dicarboxylic acids of interest were analyzed in the negative mode by MRM. The following mass transitions (m/z) were used: suberic acid, 173→111; sebacic acid-d16, 217→153; thapsic acid, 285→267. The ion spray voltage was -4,500 eV, the probe temperature was 500°C, and the dwell time was 25 ms for each analyte.

### Export of ApoB-100

HepG2 cells were grown in a 96-well plate (25,000 cells/well) and exposed to the toxicants for 24 h. The ApoB-100 concentration was measured as a surrogate of the export of triglycerides as VLDL in 100 μL of cell supernatant using the ApoB Quantikine^®^ ELISA Kit from R&D Systems (Minneapolis, MN, United States) according to the manufacturer’s protocol.

### Statistical Methods

All results are expressed as mean ± standard error of the mean (SEM). Statistical analysis and calculation of IC_50_ values were performed with the statistics program Prism 7 from GraphPad Software (La Jolla, CA, United States). Differences between groups were determined by one-way-ANOVA followed by a Dunnett post-test. *P*-values < 0.05 were considered as significant and marked with ^∗^.

## Results

We included six compounds in our study; three established inhibitors of hepatic fatty acid metabolism [etomoxir as an inhibitor of CPT1A (Lilly et al., 1992; Ceccarelli et al., 2011), MCPA as an inhibitor of medium chain acyl-CoA dehydrogenase (Ikeda and Tanaka, 1990; Tserng et al., 1991) and 4-BCA as an inhibitor of 3-keto-acyl-CoA thiolase (Olowe and Schulz, 1982)] and three test compounds known to interfere with hepatic fatty acid metabolism and to be associated with liver steatosis (amiodarone, tamoxifen, and WIN 55,212-2) (Purohit et al., 2010; Amacher, 2014) (Supplementary Figure 1). The idea was to study first the effect of the well characterized inhibitors (etomoxir, MCPA, and 4-BCA) on the assays used in order to find out whether we obtained the expected results. The experience gained in this first step could then be used to judge the results obtained by the less characterized compounds (amiodarone, tamoxifen, and WIN 55,212-2) in order to find out mechanisms how these compounds inhibit fatty acid metabolism.

### Membrane Integrity and Cellular ATP

First, we investigated plasma membrane integrity and effect on the cellular ATP content of the three established inhibitors. For that, we exposed HepG2 cells for 24 h to different concentrations of the three inhibitors. The concentration-toxicity relationships are shown in Supplementary Figure 2 and the corresponding IC_50_ values are listed in **Table 1**. All established inhibitors tested significantly decreased the cellular ATP content and impaired membrane integrity. The depletion of the cellular ATP content occurred at lower concentrations than impairment of membrane integrity, which is an indicator of mitochondrial toxicity (Kamalian et al., 2015). Etomoxir depleted the cellular ATP content most potently, whereas 4-BCA was the most potent compound regarding impairment of membrane integrity.

**Table 1:** IC_50_ values for AK release, ATP content, and β-oxidation.

	AK release	ATP content	$\frac{{\text{IC}}_{50}\text{AK}}{{\text{IC}}_{50}\text{ATP}}$	β-oxidation
Etomoxir	>200	177 ± 27	>1.1	0.02 ± 0.001
MCPA	>500	>500	n.d.	28.0 ± 4.6
4-BCA	1340 ± 410	262 ± 18	5.11	113 ± 10
Amiodarone	80.0 ± 1.0	36.1 ± 1.5	2.22	29.5 ± 3.5
Tamoxifen	24.0 ± 1.0	14.8 ± 2.1	1.62	30.4 ± 3.5
WIN 55,212-2	>10	9.2 ± 0.2	>1.1	2.0 ± 0.1

*The IC_50_ values [μM] of the compounds investigated were calculated based on the experiments shown in Supplementary Figures 2, 3 and in **Figure 2**. Calculations were performed using Prism 7 from GraphPad Software (La Jolla, CA, United States). Data are presented as are presented as mean ± SEM of at least 3 independent experiments. n.d. not determinable.*

The concentration-toxicity curves and the corresponding IC_50_ values of the three test compounds are shown in Supplementary Figure 3 and **Table 1**, respectively. All three test compounds significantly depleted the cellular ATP pool and impaired membrane integrity. Similar to the established inhibitors, the test compounds depleted the cellular ATP pool at lower concentrations than impairment of membrane integrity occurred (**Table 1**).

### Intracellular Lipid Accumulation

**FIGURE 1:**
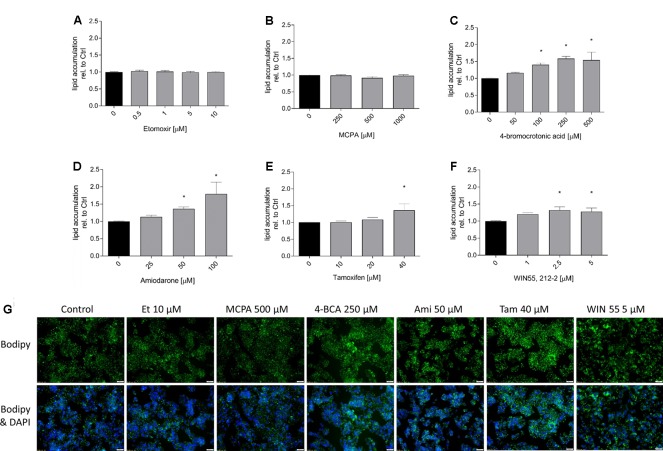
Effect on intracellular lipid accumulation in HepG2 cells. HepG2 cells were cultured in a medium containing palmitate and oleate and treated with the compounds of interest for 24 h. After treatment, cells were stained with 10 μg/mL propidium iodide and BODIPY, and eventually analyzed by FACS. **(A)** etomoxir, **(B)** MCPA, **(C)** 4-BCA, **(D)** amiodarone, **(E)** tamoxifen, and **(F)** WIN 55,212-2. Below the columns there are representative fluorescence microscopy pictures **(G)**. These HepG2 cells were treated as described above and stained with BODIPY (green for fat) and DAPI (blue for nuclei). Results were normalized to control values (DMSO 0.1%) and are presented as mean ± SEM. ^∗^*p* < 0.05 vs. DMSO 0.1% control.

In order to demonstrate the effect on hepatic fatty acid metabolism of the toxicants studied, we first investigated their potential for cellular accumulation of neutral lipids (mainly triglycerides) by staining with BODIPY 493/503. As shown in **Figure 1**, with the exception of etomoxir and MCPA, all substances tested were associated with lipid accumulation in a concentration-dependent fashion, starting at concentrations in the range of the IC_50_ values for ATP depletion. The results obtained with FACS were confirmed by the determination of lipid accumulation using the fluorescence microscope (**Figure 1G**).

### Effect on ^14^C-Palmitate Metabolism in HepG2 Cells

**FIGURE 2:**
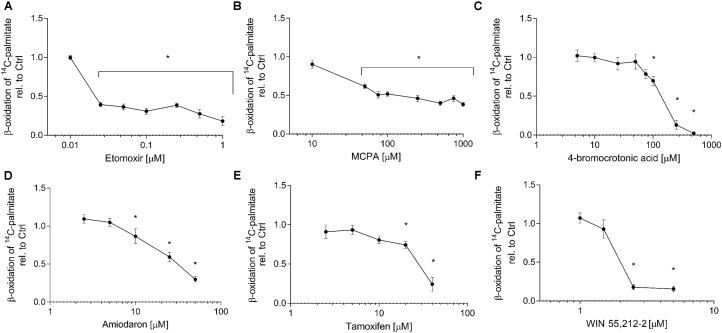
Effect on 1-^14^C-palmitate metabolism in HepG2 cells. HepG2 cells were exposed to **(A)** etomoxir, **(B)** MCPA, **(C)** 4-bromocrotonic acid, **(D)** amiodarone, **(E)** tamoxifen, or **(F)** WIN 55,212-2 for 24 h and metabolism of 1-^14^C-palmitic acid was determined in permeabilized cells. Basal rates of palmitate oxidation for control incubations (0.1% DMSO) were 5.9 ± 0.5 nmol palmitate × min^-1^ × mg protein^-1^. Results are expressed relative to DMSO 0.1% exposed control values and are presented as mean ± SEM. ^∗^*p* < 0.05 vs. DMSO 0.1% control.

The quantification of the metabolism of ^14^C-palmitate represents a specific way to assess the effect of toxicants on activation, intracellular transport and β-oxidation of long-chain fatty acids. All compounds investigated significantly inhibited palmitate metabolism. The concentration-toxicity curves are shown in **Figure 2**, and the corresponding IC_50_ values in **Table 1**. The most potent established inhibitor was etomoxir, with an IC_50_ in the nanomolar range. With the exception of tamoxifen, the IC_50_ values were either lower (etomoxir, MCPA, 4-BCA, and WIN 55,212-2) or similar (amiodarone) as the IC_50_ values for cellular ATP depletion. Similar results were obtained in isolated mouse liver mitochondria (data not shown).

### Analysis of Acylcarnitines

Another possibility to assess effects on fatty acid metabolism is by analyzing the acylcarnitine pool in the supernatant of the incubations. The cellular carnitine pool is much larger than the CoA pool and acyl-CoAs can be converted to the corresponding acylcarnitines by carnitine acyltransferases. In contrast to acyl-CoAs, acylcarnitines can leave mitochondria and cells by specific transport systems and can therefore be detected in cell supernatants and body fluids. Since acylcarnitines reflect the cellular CoA pool, they can be used for the detection and the characterization of disturbances in fatty acid metabolism (Minkler and Hoppel, 1993; Vernez et al., 2003).

**FIGURE 3:**
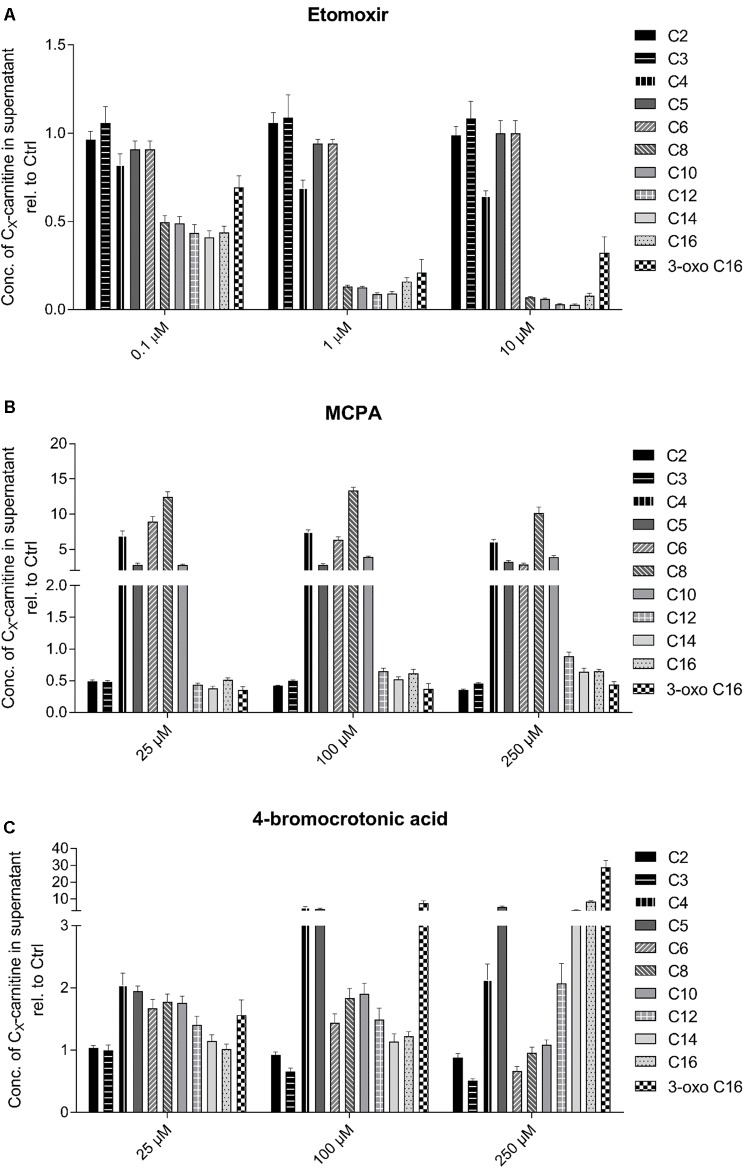
Effect of **(A)** etomoxir, **(B)** MCPA, and **(C)** 4-BCA on the acylcarnitine pattern in the supernatant of HepG2 cells. Acylcarnitines were analyzed in the supernatant of HepG2 cells treated with the compounds of interest for 24 h using LC-MS/MS as described in the section “Materials and Methods.” Results were normalized to the values obtained in DMSO 0.1% exposed control cells. Values are expressed as mean ± SEM.

As shown in **Figure 3**, the established inhibitors were associated with the predicted changes in the acylcarnitine pool of the cell supernatant. As expected, HepG2 cells exposed to increasing concentrations of the CPT1 inhibitor etomoxir showed a decrease in C8 to C16 acylcarnitines in the supernatant (**Figure 3A**). Fatty acids with less than 8 carbons can be transported independently of CPT1 into the mitochondrial matrix (Rinaldo, 2001). The medium-chain acyl-CoA dehydrogenase inhibitor MCPA was associated with a massive increase in the medium-chain acylcarnitine concentration in the cell supernatant (C4–C10 acylcarnitines, **Figure 3B**) (Zschocke et al., 2001; Okun et al., 2002). The 3-keto-acyl-CoA thiolase inhibitor 4-BCA was associated with a concentration-dependent increase in β-keto C16 acylcarnitine (**Figure 3C**).

**FIGURE 4:**
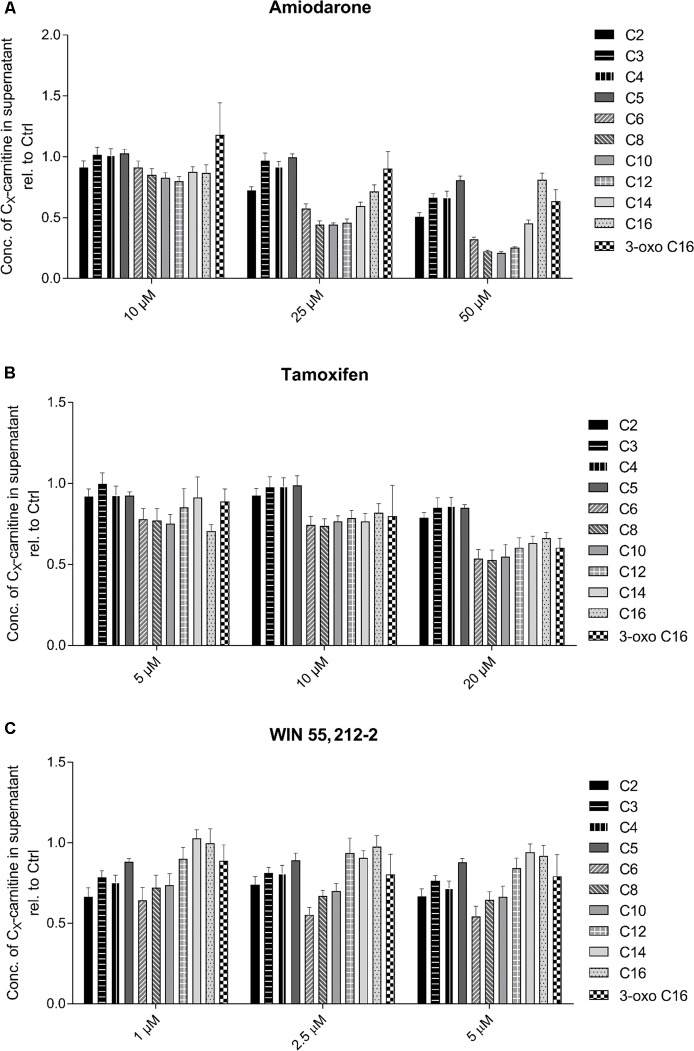
Effect of **(A)** amiodarone, **(B)** tamoxifen, and **(C)** WIN 55,212-2 on the acylcarnitine pattern in the supernatant of HepG2 cells. Acylcarnitines were analyzed in the supernatant of HepG2 cells treated with the compounds of interest for 24 h using LC-MS/MS as described in the section “Materials and Methods.” Results were normalized to the values obtained in DMSO 0.1% exposed control cells. Values are expressed as mean ± SEM.

Regarding the test compounds (**Figure 4**), amiodarone showed a clear, concentration-dependent decrease in medium-chain acylcarnitines (C6–C14) (**Figure 4A**). Also tamoxifen (**Figure 4B**) and WIN 55,212-2 (**Figure 4C**) showed concentration dependent decreases in C6–C10 acylcarnitines, but the decreases were less accentuated than those for amiodarone.

For etomoxir and tamoxifen, similar data were obtained in HepaRG cells (data not shown).

### Analysis of Dicarboxylic Acids

Acylcarnitines exported from mitochondria can also reach the endoplasmic reticulum, where they can undergo hydrolysis and ω-oxidation by for instance CYP4A11. The end product is the corresponding ω-aldehyde of the carboxylic acid, which can be oxidized to the corresponding dicarboxylic acid in peroxisomes (Wanders et al., 2011). Dicarboxylic acids of different chain lengths may therefore serve as indirect markers for inhibition of mitochondrial fatty acid metabolism. As shown in Supplementary Figure 4, we found a significant increase in the formation of thapsic acid (Supplementary Figure 4A), which is the dicarboxylic acid corresponding to palmitate, in the presence of 10 μM etomoxir. In comparison, amiodarone (50 μM) and tamoxifen (40 μM) significantly decreased the formation of thapsic acid. In contrast to thapsic acid, the formation of suberic acid (C8) was not affected by any of the toxicants investigated (Supplementary Figure 4B).

### Effect on Lipid Metabolism in Isolated Mitochondria

**FIGURE 5:**
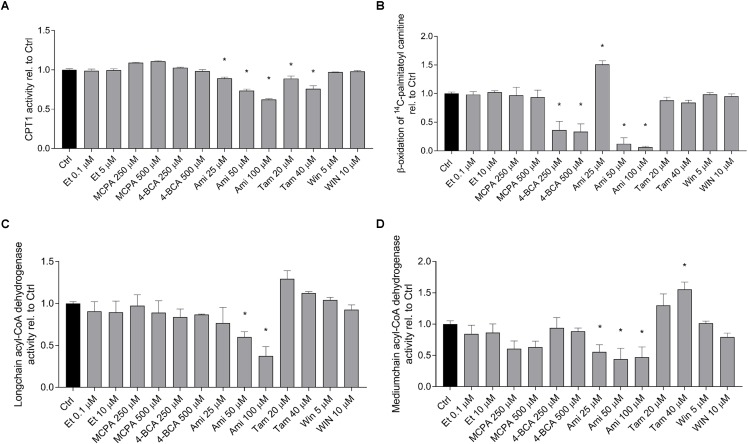
Effect on fatty acid metabolism in isolated mouse liver mitochondria. Isolated, previously frozen mouse liver mitochondria were used as enzyme source. **(A)** Effect on CPT1A activity. Formation of ^14^C-palmitoylcarnitine from ^14^C-palmitoyl-CoA was assessed after incubation with the toxicants for 10 min. Basal rates of palmitoyl-CoA formation for control incubations (0.1% DMSO) were 3.0 ± 0.25 nmol palmitoyl-CoA × min^-1^ × mg protein^-1^. **(B)** Effect on 1-^14^C-palmitoylcarnitine metabolism. Mitochondria were treated with the toxicants for 10 min before formation of acid soluble products was determined. Basal rates of palmitoylcarnitine oxidation for control incubations (0.1% DMSO) were 213 ± 22 nmol palmitoylcarnitine × min^-1^ × mg protein^-1^. **(C,D)** Effect on long-chain and medium-chain acyl-CoA dehydrogenases. After pretreatment with the toxicants for 3 min, reduction of cytochrome c by FADH produced by the acyl-CoA dehydrogenases was determined spectrophotometrically. Basal rates of cytochrome c oxidation by long- and medium-chain acyl-CoA dehydrogenases in control incubations (0.1% DMSO) were 53.7 ± 2.2 nmol × min^-1^ × mg protein^-1^ and 32.3 ± 1.2 nmol × min^-1^ × mg protein^-1^, respectively. Results were normalized to the values obtained in DMSO 0.1% exposed controls and are expressed as mean ± SEM. ^∗^*p* < 0.05 vs. DMSO 0.1% control.

In order to confirm the results obtained by quantification of palmitate metabolism and the acylcarnitine and dicarboxylic acid pools in the cell supernatant, we determined the activity of the enzymes that were predicted to be affected by the toxicants investigated in isolated and previously frozen mouse liver mitochondria. As shown in **Figure 5A**, the activity of CPT1A was significantly inhibited by amiodarone and tamoxifen in a dose-dependent manner. Etomoxir did not inhibit CPT1A under these conditions, because it inhibits CPT1 after formation of the CoA derivative and CoASH was not present in the assay. The metabolism of palmitoylcarnitine, which reflects the import of fatty acids into mitochondria as well as β-oxidation (**Figure 5B**), was significantly inhibited by 4-BCA and by the higher concentrations of amiodarone (50 and 100 μM). The activity of the long-chain acyl-CoA dehydrogenase (**Figure 5C**) was significantly inhibited by amiodarone 50 and 100 μM, and the activity of the medium-chain acyl-CoA dehydrogenase was decreased by MCPA and amiodarone (**Figure 5D**).

The results show that amiodarone and tamoxifen inhibit CPT1A (as predicted from the acylcarnitine pattern in the cell supernatant) and that amiodarone additionally inhibits long-chain and medium-chain acyl-CoA dehydrogenase.

### ApoB-100 Excretion

**FIGURE 6:**
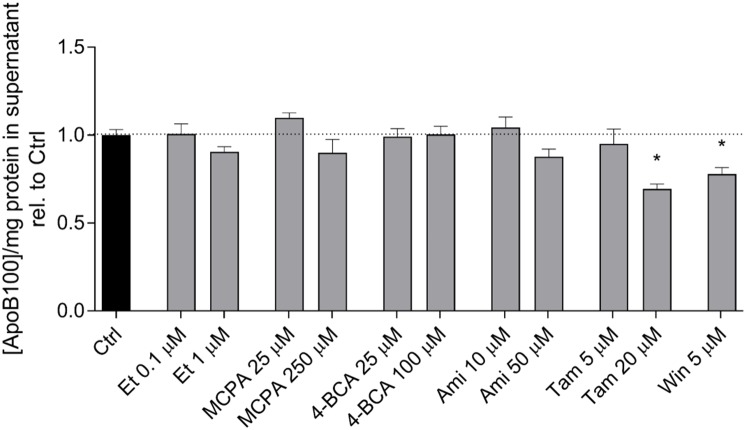
Effect on the ApoB-100 concentration in the supernatant of HepG2 cells. The ApoB concentration in the supernatant of HepG2 cells treated with the compounds of interest for 24 h was measured using an ELISA as described in the section “Materials and Methods.” The mean ApoB-100 concentration in the supernatant of control cells exposed to 0.1% DMSO was 3.10 ± 0.12 μg × mg protein^-1^. Results are normalized to control cells exposed to 0.1% DMSO and are presented as mean ± SEM. ^∗^*p* < 0.05 vs. DMSO 0.1% control. Ctrl, control; Et, etomoxir; MCPA, methylenecyclopropylacetic acid; 4-BCA, 4-bromocrotonic acid; Ami, amiodarone; Tam, tamoxifen; Win, WIN 55,212-2.

In hepatocytes, activated fatty acids can either be transported into the mitochondrial matrix for β-oxidation or be used for triglyceride synthesis. Triglycerides can be transported out of the cells in the form of VLDL. To assess VLDL excretion, we determined the concentration of ApoB-100 in the cell supernatant, which is the protein backbone of VLDL (Fisher and Ginsberg, 2002). As shown in **Figure 6**, etomoxir, MCPA, 4-BCA, and amiodarone did not affect the ApoB-100 concentration in the cell supernatant after an incubation period of 24 h. In contrast, tamoxifen (20 μM) and WIN 55,212-2 (5 μM) significantly decreased the excretion of ApoB after 24 h of treatment, suggesting inhibition of VLDL excretion.

## Discussion

The principle aims of the current study were to investigate the suitability of HepG2 cells for studying the effect of toxicants on fatty acid metabolism and to test new methods for assessing toxic effects on hepatocellular lipid metabolism, which could also provide information about the associated mechanisms. Therefore, we first investigated the effects of three established inhibitors of fatty acid metabolism; etomoxir as an inhibitor of CPT1A (Lilly et al., 1992; Ceccarelli et al., 2011), MCPA as an inhibitor of medium chain acyl-CoA dehydrogenase (Ikeda and Tanaka, 1990; Tserng et al., 1991) and 4-BCA as an inhibitor of 3-keto-acyl-CoA thiolase (Olowe and Schulz, 1982; Yao et al., 1994). In a second step, we investigated the effects of three less characterized test compounds (amiodarone, tamoxifen, and WIN 55,212-2) in order to find out if and how they inhibit fatty acid metabolism by HepG2 cells.

With the exception of etomoxir and MCPA, all compounds investigated were associated with cellular triglyceride accumulation. Regarding etomoxir and MCPA, this finding is surprising, since both compounds strongly inhibited palmitate metabolism with an IC_50_ clearly lower than the highest concentrations used in the experiments with fat accumulation. Hepatic lipid droplets are formed in the endoplasmic reticulum and have a core of triglycerides and sterol esters, which is surrounded by phospholipids. Importantly, they contain a number of proteins on their surface, which are responsible for their formation and degradation (Khan et al., 2015; Mashek et al., 2015). Regarding the complexity of the composition and the metabolism of hepatic lipid droplets, it can be imagined that inhibition of fatty acid breakdown by etomoxir and MCPA is not sufficient for lipid droplet formation. This notion is in line with the multiple parallel hit hypothesis of lipid droplet formation proposed by Tilg and Moschen (2010), which states that multiple factors are necessary for lipid droplet formation. Importantly, this means that impairment of hepatic fatty acid metabolism can be missed, if lipid droplet accumulation is used as the only assay of fatty acid metabolism.

In comparison, the determination of palmitate metabolism using ^14^C-palmitate proved to be a reliable assay for detecting an impairment of fatty acid metabolism in HepG2 cells. All compounds investigated inhibited ^14^C-palmitate metabolism at relevant concentrations. The assay is simple and works in both cells and isolated mitochondria (Krahenbuhl et al., 1994; Kaufmann et al., 2005; Felser et al., 2013). The product measured consists of water-soluble degradation compounds, which, in the case of hepatocytes and HepG2 cells, may mainly be acetyl-CoA, acetylcarnitine and ketone bodies. In permeabilized cells, the assay can detect impaired activation, transport into the mitochondrial matrix and β-oxidation. In comparison to the determination of the acylcarnitine pool in the cell supernatant, the determination of the palmitate metabolism provides no information about the location where long-chain fatty acid metabolism is inhibited.

Similar to palmitate metabolism, also the investigation of the acylcarnitine pattern in the supernatant yielded the expected results for the three established inhibitors. Based on the experience with this assay in patients with fatty acid oxidation disorders (Ensenauer et al., 2012), this result could be expected. The CPT1 inhibitor etomoxir decreased the C8 to C16 acylcarnitine concentrations due to a block of the transport of palmitate into the mitochondrial matrix (Lilly et al., 1992). The medium-chain acyl-CoA dehydrogenase inhibitor MCPA was associated with a massive increase in the C4 to C10 acylcarnitines, demonstrating the specificity of this inhibitor toward medium-chain acyl-CoA dehydrogenase. In comparison, 4-BCA showed a more complex pattern. As expected, 3-oxo-palmitate was increased, demonstrating the block of the long-chain β-ketoacyl-CoA thiolase. Since also C10 to C12 and C4 and C5 acylcarnitines were increased, the block appears not to be specific for the long-chain β-keto acyl-CoAs, but probably includes also medium- and short-chain β-ketoacyl-CoAs (Kamijo et al., 1997).

The test compounds did not show such a clear picture. Amiodarone was associated with a decrease in C6 to C16 acylcarnitines, which is compatible with CPT1A inhibition. Inhibition of CPT1A by amiodarone was shown directly in the current study using isolated liver mitochondria (**Figure 5**) and has been described previously in rat liver (Kennedy et al., 1996). Interestingly, C14 and C16 acylcarnitine concentrations were increased compared to C6 to C12 acylcarnitines and also compared to the CPT1A inhibitor etomoxir, suggesting that amiodarone inhibited β-oxidation of long-chain fatty acids in addition to CPT1A. This was confirmed directly in isolated mitochondria where amiodarone inhibited the long- and medium-chain acyl-CoA dehydrogenase (**Figure 5**).

Tamoxifen showed a decrease in C6 to C16 acylcarnitines, compatible with an inhibition of the import of long-chain fatty acids into the mitochondrial matrix. This was confirmed by inhibition of CPT1A in isolated liver mitochondria (**Figure 5**). Finally, WIN 55,212-2 exhibited a decrease of C2 to C10 acylcarnitines but normal concentrations of C12 to C16 acylcarnitines. Similar to amiodarone, this pattern could have been explained by impaired mitochondrial import of long-chain fatty acids combined with impaired β-oxidation. However, in contrast to amiodarone and tamoxifen, WIN 55,212-2 did not impair the function of CPT1A or of β-oxidation (**Figure 5**). The mechanism by which WIN 55,212-2 inhibits palmitate metabolism could therefore not be explained by the assays used in the current study. Inhibition of fatty acid activation is a possibility that we did not test.

Acylcarnitines cannot only be excreted, but can also be converted to the corresponding dicarboxylic acids by the ω-oxidation pathway, which involves members of microsomal enzymes of the CYP4A- and 4F-family as well as the peroxisomal fatty aldehyde dehydrogenase (FALHD) (Kelson et al., 1997; Wanders et al., 2011). Dicarboxylic acids are therefore potential biomarkers for impaired mitochondrial fatty acid metabolism that could also be used *in vivo*. Etomoxir was the only compound of the toxicants studied that caused an increase in thapsic (C16 dicarboxylic) acid in the cell supernatant. The finding that CPT1A inhibitors can induce the formation of dicarboxylic acids has been described previously and has been explained by induction of CYP4A1, a microsomal enzyme responsible for ω-oxidation of fatty acids (Kaikaus et al., 1993). Interestingly, amiodarone and tamoxifen inhibited the formation of thapsic acid (Supplementary Figure 4). At least amiodarone and its N-deethylated metabolites are known CYP inhibitors (Ohyama et al., 2000), which may explain this finding.

The excretion of ApoB-100 was determined as a marker of the excretion of VLDL, which is a lipoprotein rich in triglycerides (Tiwari and Siddiqi, 2012). Both tamoxifen and WIN 55,212-2 inhibited the excretion of ApoB-100, whereas the other compounds had no significant effect. The steatogenic effect of tamoxifen has been attributed in previous studies to increased fatty acid synthesis (Cole et al., 2010; Zhao et al., 2014) and impaired fatty acid β-oxidation (Tuquet et al., 2000; Zhou et al., 2012). The results of the current study are in agreement with these studies and additionally suggest that tamoxifen also affects VLDL secretion. This has recently been demonstrated directly in a study in humans (Birzniece et al., 2017). In comparison, no mechanistic studies are so far available for WIN 55,212-2. Studies for other cannabinoids suggest, that the steatogenic effect of these compounds is due to stimulation of the CB1 receptors, which is associated with a downregulation of CPT1 expression and an increased expression of the transcription factor SREBP-1c (Purohit et al., 2010; Regnell, 2013; Zhang et al., 2014). The results of the current study suggest that WIN 55,212-2 affects both fatty acid breakdown and triglyceride excretion.

The current study has strengths and weaknesses. A strength is certainly that most alterations in the acylcarnitine patterns were confirmed by determination of the respective enzyme activities in isolated mouse liver mitochondria. A weakness is that we did not use primary human hepatocytes for comparison of the findings in HepG2 cells. Since we obtained the predicted results for the established inhibitors in HepG2 cells and since we confirmed the most important findings in mouse liver mitochondria, we believe that the results obtained in HepG2 cells are reliable. In support of this statement, HepG2 cells have been proposed as an *in vitro* model for assessing hepatocellular fatty acid metabolism by other researchers (Amacher, 2011; Donato and Gomez-Lechon, 2012).

## Conclusion

The current study shows that amiodarone impairs fatty acid metabolism by inhibiting CPT1A and long- and medium-chain acyl-CoA dehydrogenase, whereas tamoxifen and WIN 55,212-2 inhibit fatty acid degradation and ApoB-100 excretion, compatible with impaired VLDL excretion. HepG2 cells are a suitable human cell line for studying certain aspects of fatty acid metabolism *in vitro*. The determination of the acylcarnitine pool provides valuable information about the location of inhibition of fatty acid degradation and may be a useful *in vivo* biomarker in future studies.

## Author Contributions

DG: data generation, data interpretation, and writing manuscript. UD: data generation, data interpretation, and correction of final manuscript. SK: study design, data interpretation, and writing of manuscript.

## Conflict of Interest Statement

The authors declare that the research was conducted in the absence of any commercial or financial relationships that could be construed as a potential conflict of interest.
