# Supplementary material for: Effect of Toxicants on Fatty Acid Metabolism in HepG2 Cells
Source: Front Pharmacol. 2018 Apr 23;9:257. doi: 10.3389/fphar.2018.00257 (PMC5924803; doi:10.3389/fphar.2018.00257)
Supplement: Supplementary file 1 [file Presentation_1.PDF]

Legends to supplementary Figures

Supplementary Figure 1

Chemical structures of etomoxir, methylenecyclopropylacetic acid (MCPA), 4-bromocrotonic acid (4-BCA), amiodarone, tamoxifen, and WIN55,212-2.

Supplementary Figure 2

Effect of etomoxir, methylenecyclopropylacetic acid (MCPA) and 4-bromocrotonic acid (4-BCA) on plasma membrane integrity and on the cellular ATP content. HepG2 cells were exposed to the toxicants for 24 h. Membrane integrity is expressed as the percentage of dead cells with Triton X set at 100%. ATP content is displayed as the percentage in relation to control incubations (DMSO 0.1%) set at 100%. (**A,B**) etomoxir, (**C,D**) MCPA, (**E,F**) 4-BCA. Results are given as mean ± SEM in relation to control incubations (DMSO 0.1%).*$p<0.05$ vs. DMSO 0.1% control.

Supplementary Figure 3

Effect of amiodarone, tamoxifen and WIN55,212-2 on plasma membrane integrity and on the cellular ATP content. HepG2 cells were exposed to the compounds of interest for 24 h. Membrane integrity is expressed as the percentage of dead cells with Triton X set at 100%. ATP content is displayed as the percentage in relation to control incubations (DMSO 0.1%) set at 100%. (**A,B**) amiodarone, (**C,D**) tamoxifen, (**E,F**) 4-WIN55,212-2. Results are given as mean ± SEM in relation to control incubations (DMSO 0.1%).*$p<0.05$ vs. DMSO 0.1% control.

Supplementary Figure 4

Effect on the dicarboxylic acid concentration in HepG2 cells. Dicarboxylic acids were analyzed in HepG2 cells treated with the compounds of interest for 24 h using LC-MS/MS as described in Methods. Results were normalized to the values obtained in DMSO 0.1%

exposed control cells. Values are expressed as mean ± SEM. *$p<0.05$ vs. DMSO 0.1% control.
