# Supplementary figures and images for: Effect of Toxicants on Fatty Acid Metabolism in HepG2 Cells
Source: Front Pharmacol. 2018 Apr 23;9:257. doi: 10.3389/fphar.2018.00257 (PMC5924803; doi:10.3389/fphar.2018.00257)

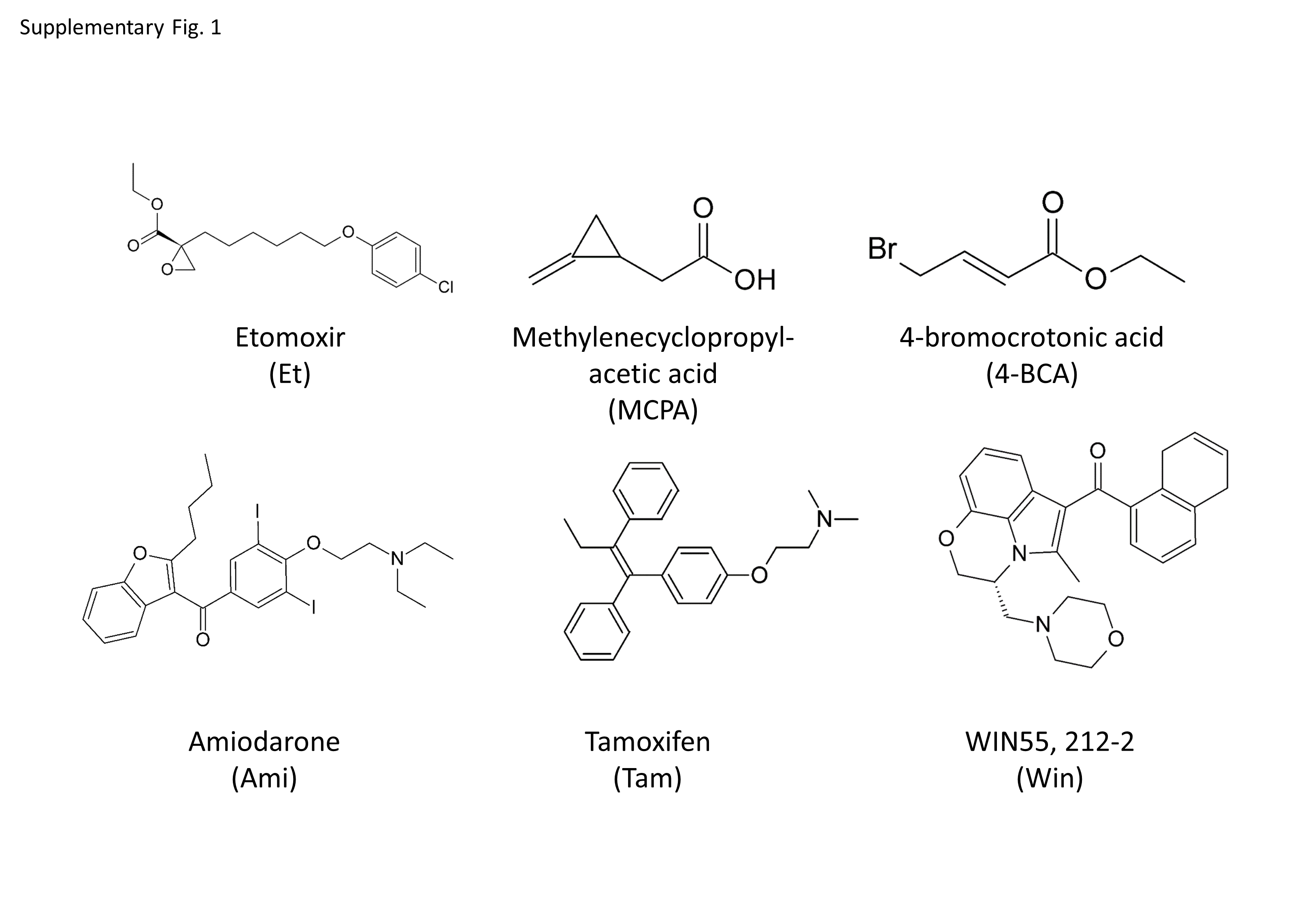

Supplement: Supplementary file 2 [file Image_1.TIF]

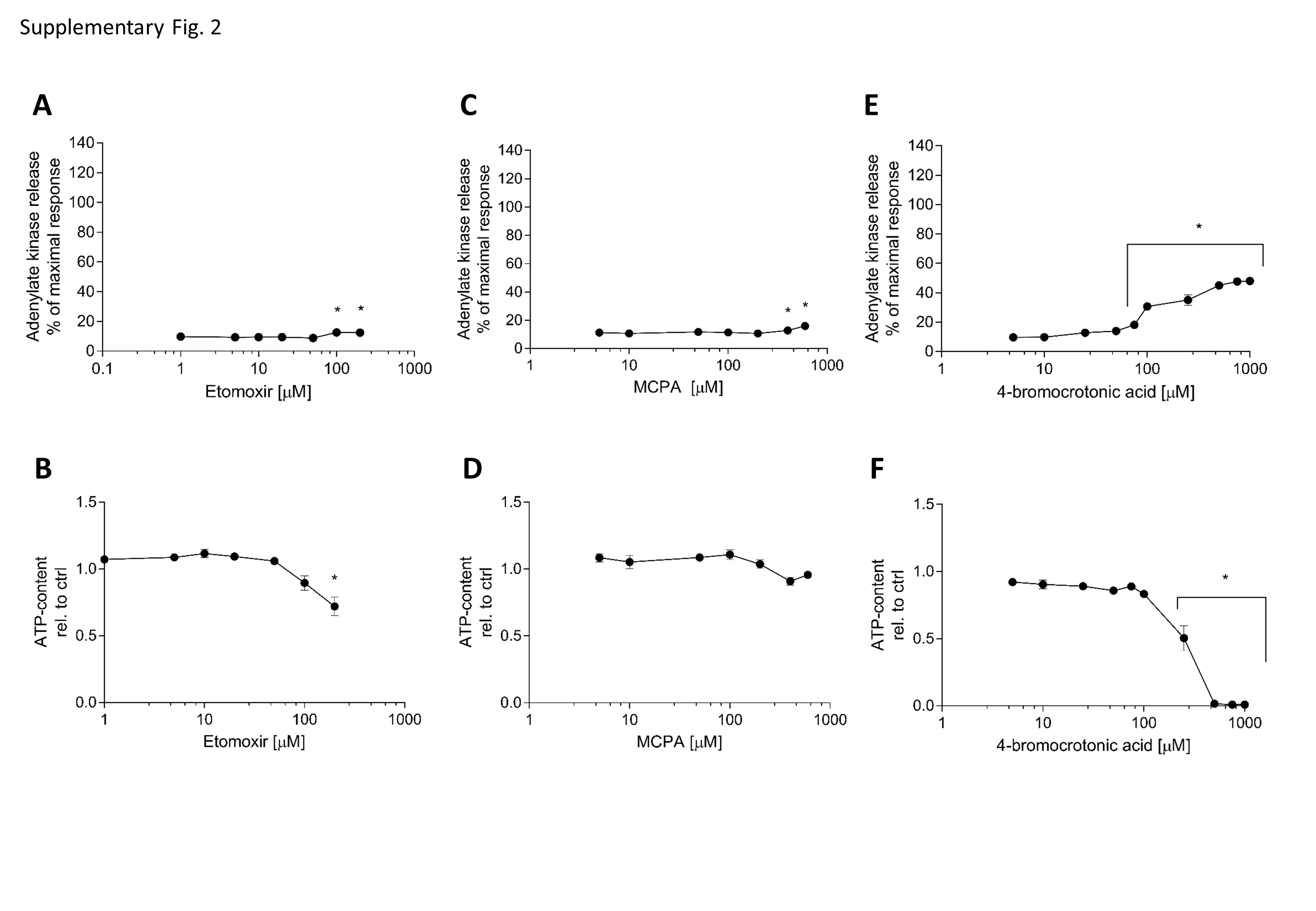

Supplement: Supplementary file 3 [file Image_2.TIF]

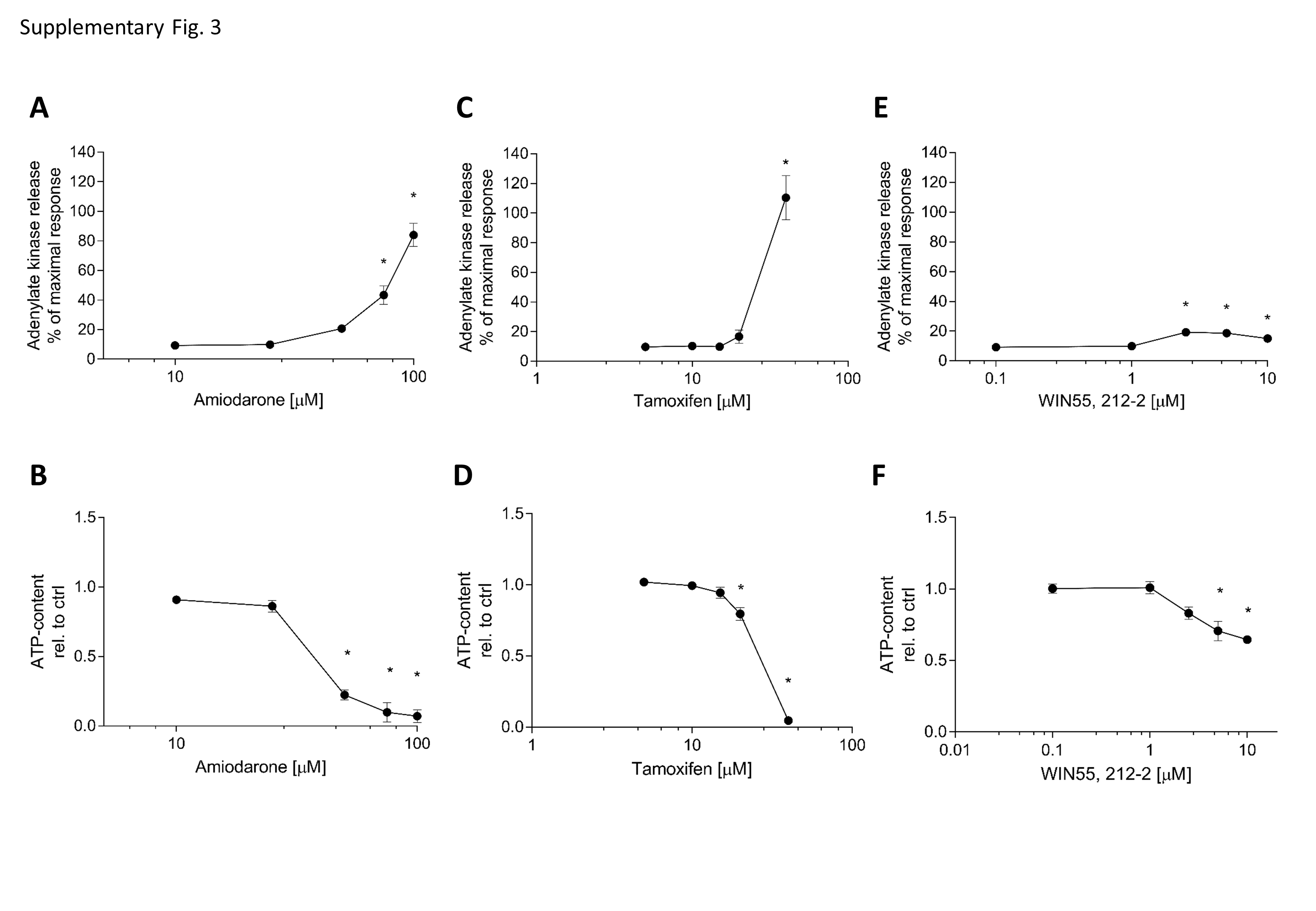

Supplement: Supplementary file 4 [file Image_3.TIF]

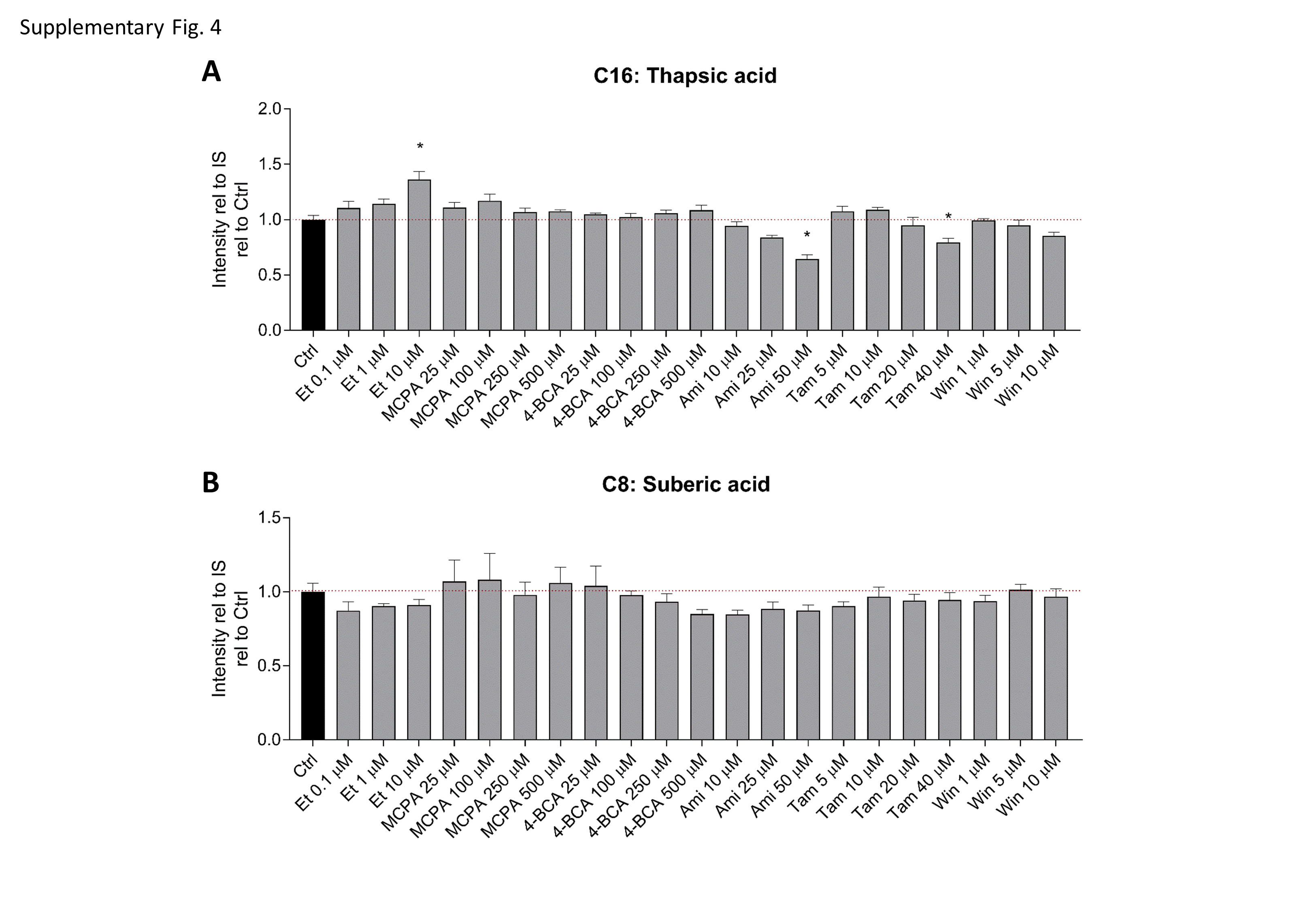

Supplement: Supplementary file 5 [file Image_4.TIF]
